# 2,4,6-Trimethyl­anilinium bromide

**DOI:** 10.1107/S1600536809034497

**Published:** 2009-09-09

**Authors:** Li-Jing Cui, Hai-Jun Xu

**Affiliations:** a Ordered Matter Science Research Center, College of Chemistry and Chemical Engineering, Southeast University, Nanjing 211189, People’s Republic of China

## Abstract

In the title compound, C_9_H_14_N^+^·Br^−^, an intra­molecular N—H⋯Br inter­action links the anion to the cation. In the crystal structure, inter­molecular N—H⋯Br inter­actions link the mol­ecules into a three-dimensional network.

## Related literature

For related structures, see: Lemmerer & Billing (2007 ▶); Long *et al.* (2007 ▶).
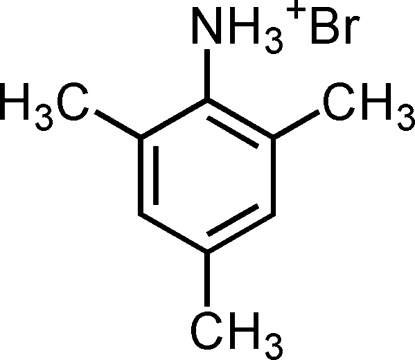


## Experimental

### 

#### Crystal data

C_9_H_14_N^+^·Br^−^
*M* *_r_* = 216.11
Orthorhombic, 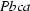

*a* = 10.399 (2) Å
*b* = 18.720 (4) Å
*c* = 10.282 (2) Å
*V* = 2001.6 (7) Å^3^
*Z* = 8
Mo *K*α radiation
μ = 4.05 mm^−1^
*T* = 294 K
0.2 × 0.2 × 0.2 mm

#### Data collection

Rigaku SCXmini diffractometer
Absorption correction: multi-scan (*CrystalClear*; Rigaku, 2005 ▶) *T*_min_ = 0.88, *T*_max_ = 1.000
18944 measured reflections
2292 independent reflections
1627 reflections with *I* > 2σ(*I*)
*R*_int_ = 0.099

#### Refinement

*R*[*F* ^2^ > 2σ(*F* ^2^)] = 0.050
*wR*(*F* ^2^) = 0.122
*S* = 1.00
2292 reflections
113 parameters
H atoms treated by a mixture of independent and constrained refinement
Δρ_max_ = 0.37 e Å^−3^
Δρ_min_ = −0.41 e Å^−3^

### 

Data collection: *CrystalClear* (Rigaku, 2005 ▶); cell refinement: *CrystalClear*; data reduction: *CrystalClear*; program(s) used to solve structure: *SHELXS97* (Sheldrick, 2008 ▶); program(s) used to refine structure: *SHELXL97* (Sheldrick, 2008 ▶); molecular graphics: *SHELXTL* (Sheldrick, 2008 ▶); software used to prepare material for publication: *SHELXL97*.

## Supplementary Material

Crystal structure: contains datablocks I, global. DOI: 10.1107/S1600536809034497/hk2763sup1.cif

Structure factors: contains datablocks I. DOI: 10.1107/S1600536809034497/hk2763Isup2.hkl

Additional supplementary materials:  crystallographic information; 3D view; checkCIF report

## Figures and Tables

**Table 1:** Hydrogen-bond geometry (Å, °)

*D*—H⋯*A*	*D*—H	H⋯*A*	*D*⋯*A*	*D*—H⋯*A*
N1—H1*A*⋯Br1^i^	0.84 (5)	2.58 (5)	3.376 (4)	157 (4)
N1—H1*B*⋯Br1	0.94 (6)	2.47 (6)	3.391 (4)	168 (4)
N1—H1*C*⋯Br1^ii^	1.01 (5)	2.32 (5)	3.292 (4)	162 (4)

Symmetry codes: (i) 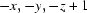; (ii) 
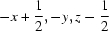
.

**Fig. 1.**
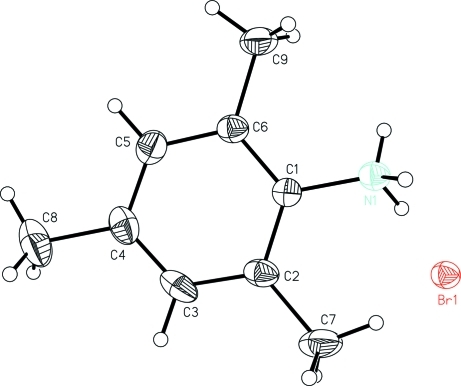
The molecular structure of the title molecule, with the atom-numbering scheme. Hydrogen bond is shown as dashed line.

## supplementary crystallographic information

### Comment

The crystal structure of the title compound is reported herein as part of a study of 2,4,6-trimethylanilinium halide salts. The other halide salts have been reported, previously (Lemmerer & Billing, 2007; Long *et al.*, 2007).

The asymmetric unit of the title compound, (Fig. 1), contains one 2,4,6 -trimethylbenzenaminium cation and one bromide anion. The intramolecular N-H···Br interaction (Table 1) links the anion to the cation.

In the crystal structure, intra- and intermolecular N-H···Br interactions (Table 1) link the molecules into a three-dimensional network.

### Experimental

For the preparation of the title compound, 2,4,6-trimethylaniline (3 mmol) was dissolved in ethanol (6 ml), and concentrated hydrobromic acid was added dropwise to dissolve the solid phase persisting in a mixture of bismuth tricbromide (3 mmol) and water (5 ml). The two solutions were then mixed and stirred for 15 min. The resulting precipitate was filtered off and dissolved in hydrobromic acid. Colorless crystals suitable for X-ray analysis were formed after several weeks by slow evaporation of the solvent at room temperature.

### Refinement

Atoms H1A, H1B and H1C (for NH_3_) are located in a difference Fourier map and refined isotropically. The remaining H atoms were positioned geometrically with C-H = 0.93 and 0.96 Å for aromatic and methyl H atoms, respectively, and constrained to ride on their parent atoms, with U_iso_(H) = xU_eq_(C), where x = 1.5 for methyl H and x = 1.2 for aromatic H atoms.

### Crystal data

**Table:**

C_9_H_14_N^+^·Br^−^	*F*(000) = 880
*M**_r_* = 216.11	*D*_x_ = 1.434 Mg m^−^^3^
Orthorhombic, *P**b**c**a*	Mo *K*α radiation, λ = 0.71073 Å
Hall symbol: -P 2ac 2ab	Cell parameters from 1647 reflections
*a* = 10.399 (2) Å	θ = 3.0–27.6°
*b* = 18.720 (4) Å	µ = 4.05 mm^−^^1^
*c* = 10.282 (2) Å	*T* = 294 K
*V* = 2001.6 (7) Å^3^	Prism, colorless
*Z* = 8	0.2 × 0.2 × 0.2 mm

### Data collection

**Table:**

Rigaku SCXmini diffractometer	2292 independent reflections
Radiation source: fine-focus sealed tube	1627 reflections with *I* > 2σ(*I*)
graphite	*R*_int_ = 0.099
Detector resolution: 13.6612 pixels mm^-1^	θ_max_ = 27.5°, θ_min_ = 3.0°
ω scans	*h* = −13→13
Absorption correction: multi-scan (*CrystalClear*; Rigaku, 2005)	*k* = −24→24
*T*_min_ = 0.88, *T*_max_ = 1.000	*l* = −13→13
18944 measured reflections	

### Refinement

**Table:**

Refinement on *F*^2^	Secondary atom site location: difference Fourier map
Least-squares matrix: full	Hydrogen site location: inferred from neighbouring sites
*R*[*F*^2^ > 2σ(*F*^2^)] = 0.050	H atoms treated by a mixture of independent and constrained refinement
*wR*(*F*^2^) = 0.122	*w* = 1/[σ^2^(*F*_o_^2^) + (0.0437*P*)^2^ + 2.8318*P*] where *P* = (*F*_o_^2^ + 2*F*_c_^2^)/3
*S* = 1.00	(Δ/σ)_max_ < 0.001
2292 reflections	Δρ_max_ = 0.37 e Å^−^^3^
113 parameters	Δρ_min_ = −0.41 e Å^−^^3^
0 restraints	Extinction correction: *SHELXL97* (Sheldrick, 2008), Fc^*^=kFc[1+0.001xFc^2^λ^3^/sin(2θ)]^-1/4^
Primary atom site location: structure-invariant direct methods	Extinction coefficient: 0.0097 (8)

### Special details

**Table:**

Geometry. All e.s.d.'s (except the e.s.d. in the dihedral angle between two l.s. planes) are estimated using the full covariance matrix. The cell e.s.d.'s are taken into account individually in the estimation of e.s.d.'s in distances, angles and torsion angles; correlations between e.s.d.'s in cell parameters are only used when they are defined by crystal symmetry. An approximate (isotropic) treatment of cell e.s.d.'s is used for estimating e.s.d.'s involving l.s. planes.
Refinement. Refinement of *F*^2^ against ALL reflections. The weighted *R*-factor *wR* and goodness of fit *S* are based on *F*^2^, conventional *R*-factors *R* are based on *F*, with *F* set to zero for negative *F*^2^. The threshold expression of *F*^2^ > σ(*F*^2^) is used only for calculating *R*-factors(gt) *etc*. and is not relevant to the choice of reflections for refinement. *R*-factors based on *F*^2^ are statistically about twice as large as those based on *F*, and *R*- factors based on ALL data will be even larger.

### Fractional atomic coordinates and isotropic or equivalent isotropic displacement parameters (Å^2^)

**Table:**

	*x*	*y*	*z*	*U*_iso_*/*U*_eq_	
Br1	0.10008 (4)	0.00542 (2)	0.69604 (4)	0.0471 (2)	
N1	0.1859 (4)	0.0634 (2)	0.3959 (4)	0.0420 (9)	
H1A	0.113 (4)	0.058 (2)	0.361 (5)	0.053 (14)*	
H1B	0.171 (5)	0.042 (3)	0.477 (6)	0.085 (18)*	
H1C	0.249 (4)	0.033 (3)	0.346 (4)	0.052 (12)*	
C1	0.2218 (4)	0.1388 (2)	0.4055 (4)	0.0364 (9)	
C2	0.3168 (4)	0.1578 (2)	0.4945 (4)	0.0466 (11)	
C3	0.3457 (4)	0.2299 (3)	0.5046 (4)	0.0563 (13)	
H3A	0.4063	0.2440	0.5659	0.068*	
C4	0.2896 (5)	0.2813 (3)	0.4289 (5)	0.0545 (12)	
C5	0.2005 (4)	0.2595 (2)	0.3376 (5)	0.0523 (11)	
H5A	0.1630	0.2935	0.2837	0.063*	
C6	0.1650 (4)	0.1883 (2)	0.3237 (4)	0.0416 (10)	
C7	0.3892 (4)	0.1028 (3)	0.5721 (5)	0.0747 (17)	
H7A	0.3565	0.0561	0.5525	0.112*	
H7B	0.3786	0.1123	0.6633	0.112*	
H7C	0.4789	0.1050	0.5503	0.112*	
C8	0.3257 (6)	0.3597 (3)	0.4419 (6)	0.0892 (19)	
H8A	0.2766	0.3874	0.3811	0.134*	
H8B	0.4157	0.3655	0.4240	0.134*	
H8C	0.3077	0.3756	0.5287	0.134*	
C9	0.0696 (5)	0.1680 (3)	0.2198 (5)	0.0656 (14)	
H9A	0.0422	0.2102	0.1744	0.098*	
H9B	−0.0035	0.1454	0.2592	0.098*	
H9C	0.1093	0.1356	0.1596	0.098*	

### Atomic displacement parameters (Å^2^)

**Table:**

	*U*^11^	*U*^22^	*U*^33^	*U*^12^	*U*^13^	*U*^23^
Br1	0.0419 (3)	0.0519 (3)	0.0473 (3)	−0.00391 (19)	−0.00179 (18)	0.0025 (2)
N1	0.035 (2)	0.048 (2)	0.043 (2)	−0.0016 (17)	−0.0005 (18)	0.0009 (18)
C1	0.033 (2)	0.041 (2)	0.035 (2)	−0.0008 (17)	0.0048 (17)	0.0004 (18)
C2	0.037 (2)	0.065 (3)	0.038 (2)	−0.011 (2)	−0.0016 (19)	0.005 (2)
C3	0.052 (3)	0.078 (3)	0.039 (3)	−0.027 (3)	0.001 (2)	−0.012 (2)
C4	0.061 (3)	0.050 (3)	0.053 (3)	−0.016 (2)	0.012 (2)	−0.006 (2)
C5	0.050 (2)	0.049 (3)	0.058 (3)	0.001 (2)	0.002 (2)	0.010 (2)
C6	0.035 (2)	0.050 (2)	0.040 (2)	−0.0016 (19)	−0.0005 (18)	0.0021 (19)
C7	0.050 (3)	0.100 (4)	0.074 (4)	−0.025 (3)	−0.024 (3)	0.037 (3)
C8	0.108 (5)	0.059 (3)	0.101 (5)	−0.031 (3)	0.005 (4)	−0.016 (3)
C9	0.060 (3)	0.078 (3)	0.058 (3)	−0.018 (3)	−0.024 (2)	0.018 (3)

### Geometric parameters (Å, °)

**Table:**

N1—C1	1.464 (5)	C6—C1	1.383 (5)
N1—H1A	0.84 (5)	C6—C5	1.391 (6)
N1—H1B	0.94 (6)	C6—C9	1.507 (6)
N1—H1C	1.01 (5)	C7—H7A	0.9600
C2—C3	1.387 (6)	C7—H7B	0.9600
C2—C1	1.393 (5)	C7—H7C	0.9600
C2—C7	1.504 (6)	C8—H8A	0.9600
C3—H3A	0.9300	C8—H8B	0.9600
C4—C3	1.368 (6)	C8—H8C	0.9600
C4—C8	1.521 (7)	C9—H9A	0.9600
C5—C4	1.380 (6)	C9—H9B	0.9600
C5—H5A	0.9300	C9—H9C	0.9600
			
C1—N1—H1A	112 (3)	C1—C6—C5	117.8 (4)
C1—N1—H1B	114 (3)	C1—C6—C9	122.9 (4)
C1—N1—H1C	114 (3)	C5—C6—C9	119.3 (4)
H1A—N1—H1B	100 (4)	C2—C7—H7A	109.5
H1A—N1—H1C	108 (4)	C2—C7—H7B	109.5
H1B—N1—H1C	108 (4)	C2—C7—H7C	109.5
C2—C1—N1	118.1 (4)	H7A—C7—H7B	109.5
C6—C1—N1	119.7 (4)	H7A—C7—H7C	109.5
C6—C1—C2	122.1 (4)	H7B—C7—H7C	109.5
C1—C2—C7	122.0 (4)	C4—C8—H8A	109.5
C3—C2—C1	116.8 (4)	C4—C8—H8B	109.5
C3—C2—C7	121.1 (4)	C4—C8—H8C	109.5
C2—C3—H3A	118.3	H8A—C8—H8B	109.5
C4—C3—C2	123.3 (4)	H8A—C8—H8C	109.5
C4—C3—H3A	118.3	H8B—C8—H8C	109.5
C3—C4—C5	117.8 (4)	C6—C9—H9A	109.5
C3—C4—C8	121.5 (5)	C6—C9—H9B	109.5
C5—C4—C8	120.7 (5)	C6—C9—H9C	109.5
C4—C5—C6	122.0 (4)	H9A—C9—H9B	109.5
C4—C5—H5A	119.0	H9A—C9—H9C	109.5
C6—C5—H5A	119.0	H9B—C9—H9C	109.5

### Hydrogen-bond geometry (Å, °)

**Table:**

*D*—H···*A*	*D*—H	H···*A*	*D*···*A*	*D*—H···*A*
N1—H1A···Br1^i^	0.84 (5)	2.58 (5)	3.376 (4)	157 (4)
N1—H1B···Br1	0.94 (6)	2.47 (6)	3.391 (4)	168 (4)
N1—H1C···Br1^ii^	1.01 (5)	2.32 (5)	3.292 (4)	162 (4)

Symmetry codes: (i) −*x*, −*y*, −*z*+1; (ii) −*x*+1/2, −*y*, *z*−1/2.
